# Single-molecule study of full-length NaChBac by planar lipid bilayer recording

**DOI:** 10.1371/journal.pone.0188861

**Published:** 2017-11-30

**Authors:** Andrew Jo, Hiofan Hoi, Hang Zhou, Manisha Gupta, Carlo D. Montemagno

**Affiliations:** 1 Department of Chemical and Materials Engineering, University of Alberta, Edmonton, Alberta, Canada; 2 Ingenuity Lab, Edmonton, Alberta, Canada; 3 Department of Electrical and Computer Engineering, University of Alberta, Edmonton, Alberta, Canada; 4 National Institute for Nanotechnology, Edmonton, Alberta, Canada; Duke University, UNITED STATES

## Abstract

Planar lipid bilayer device, alternatively known as BLM, is a powerful tool to study functional properties of conducting membrane proteins such as ion channels and porins. In this work, we used BLM to study the prokaryotic voltage-gated sodium channel (Na_v_) NaChBac in a well-defined membrane environment. Na_v_s are an essential component for the generation and propagation of electric signals in excitable cells. The successes in the biochemical, biophysical and crystallographic studies on prokaryotic Na_v_s in recent years has greatly promoted the understanding of the molecular mechanism that underlies these proteins and their eukaryotic counterparts. In this work, we investigated the single-molecule conductance and ionic selectivity behavior of NaChBac. Purified NaChBac protein was first reconstituted into lipid vesicles, which is subsequently incorporated into planar lipid bilayer by fusion. At single-molecule level, we were able to observe three distinct long-lived conductance sub-states of NaChBac. Change in the membrane potential switches on the channel mainly by increasing its opening probability. In addition, we found that individual NaChBac has similar permeability for Na^+^, K^+^, and Ca^2+^. The single-molecule behavior of the full-length protein is essentially highly stochastic. Our results show that planar lipid bilayer device can be used to study purified ion channels at single-molecule level in an artificial environment, and such studies can reveal new protein properties that are otherwise not observable in *in vivo* ensemble studies.

## Introduction

**Fig 1:**
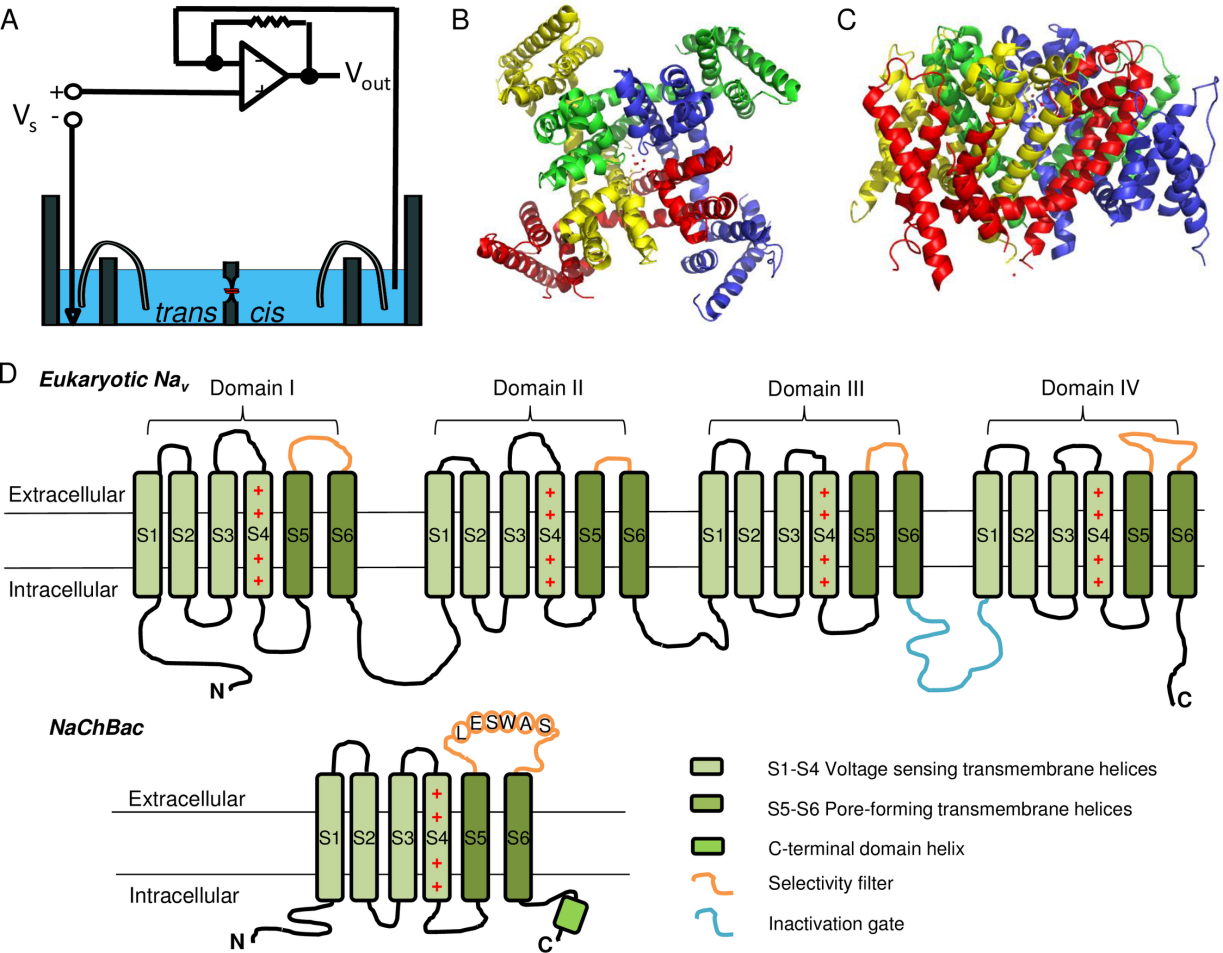
Schematic representation of the BLM setup and the structure of prokaryotic Na_v_. (A) The BLM sample chamber and the amplifier. The reference electrode is connected to the *trans* chamber through a salt bridge and the command electrode is connected to the *cis* chamber. (B) Top-view of the structure of a full-length prokaryotic Na_v_. The four monomers, highlighted in different colors, assemble along each other to form the ionic conducting pore in the center of the tetrameric structure. Picture is generated based on the X-ray crystal structure of Na_v_Rh (PDB ID 4DXW) [7]. (C) Side-view of the same structure in (B). (D) Schematic illustration comparing the structure of eukaryotic and prokaryotic Na_v_s. The four arginine residues on S4 that are responsible for the voltage sensing are denoted by “+” signs. N and C denote the N- and C- termini, respectively.

Planar lipid bilayer device, alternatively known as black lipid membrane (BLM), is an attractive technique for characterization of channel-forming proteins such as ion channels and porins [1–3]. First demonstrated in the 1960s [4], the BLM technique involved the formation of a planar lipid bilayer across an orifice by lipid painting [4] or by folding lipid monolayers into bilayer [5]. Isolated membrane protein was then inserted into the bilayer [2, 3]. Subsequently, a transmembrane voltage is applied to induce an ionic current through the protein of interest (Fig 1A). Functional characterization, mostly electrophysical, of the protein can then be interpreted. The technique was initially limited to physiochemical studies of lipid membrane due to the lack of understanding on membrane proteins and their isolation at the time. With the recent advancement in membrane protein biotechnology, there is an increasing interest in revisiting this old method for channel protein characterization. Since purified protein was used in BLM, potential influence from other proteins in the native membrane can be excluded from the recording and the protein of interest is the sole subject under study [2, 3, 6]. In addition, membrane composition can be readily controlled to study the lipid-protein interaction. The technique is also less skill-demanding than the patch clamp which has been widely used to study ion channels heterologously overexpressed in live cells [6]. BLM is thus particularly useful for studies of conductance, sub-conductance states, transition rates between states, ion selectivity, ionic strength dependence and inhibitor binding behavior for ion channels.

In this work, we applied BLM to undertake a single-channel study on NaChBac, which is a bacterial voltage-gated Na^+^ channel (Na_v_) isolated from *Bacillus halodurans* [8]. Na_v_s are a sub-family of ion channels that selectively transport Na^+^ under the regulation of membrane potential. In vertebrates, Na_v_s are responsible for the initiation and the rising phase of the action potential in excitable cells such as neurons and muscle cells [9]. The influx of Na^+^ causes a change in the membrane potential within a timescale of milliseconds and initiates downstream electrical signaling cascade. Na_v_s are important for various physiological events. Malfunction of Na_v_s has been associated with a range of disease including cardiac arrhythmias, movement disorders, pain, migraine and epilepsy [9]. Studying these channels not only provides insight into their biological roles, but it also facilitates the development of pharmaceuticals that target these proteins. However, vertebrate Na_v_s are complex membrane proteins that are composed of multiple subunits, each in turn has multiple domains [10]. Biophysical studies of these vertebrate Na_v_s have largely been daunting because of their complicated structure and the challenge to obtain enough quantity of pure functional protein. Fortunately, their prokaryotic counterparts have a much simpler structure, as they are homotetramers of a single subunit whose structure resembles one of the four domains of a vertebrate Na_v_ [11] (Fig 1B–1D, and S1 Fig). Functional characterization of prokaryotic Na_v_s suggests that they share many important features associated with canonical vertebrate Na_v_s and Ca_v_s such as voltage-dependent activation, slow activation, ion selectivity, and drug block [8, 11]. With a simpler structure, prokaryotic Na_v_s are easier to handle than vertebrate Na_v_s. Indeed, high-resolution X-ray crystal structure has been resolved for several prokaryotic Na_v_s [7, 11, 12], providing important insight into the functional mechanism of the protein family. Studying the structural and molecular basis for their function is expected to provide a model system to study the more complicated vertebrate voltage-gated ion channels.

BLM has been applied to study the conducting and inhibitor sensitivity of a variety of purified ion channels including the voltage-gated potassium channels K_v_AP [13] and KcsA [14], NaChBac [15], and the pore-only domains of the prokaryotic Na_v_SP [16, 17]. Among them, Studer and coworkers were able to use the nystatin-ergosterol method [18] to insert NaChBac into the preformed lipid bilayer and measured the unitary conductance of NaChBac by macroscopic current recording [15]. Generally, in order to achieve single-channel recording on BLM, the protein of interest is preferred to have intrinsic low activity or addition of an inhibitor is used to reduce the activity, so that activity from one single molecule is under observation at a time. We observed moderate activity for NaChBac in our lab, but we managed to achieve single channel recording by reducing the number of protein incorporated into the lipid bilayer.

## Materials and methods

### General materials

Chemicals are obtained from Sigma-Aldrich unless stated otherwise. Lipids are obtained from Avanti Polar Lipids (Alabaster, AL, USA).

### Protein purification

The cDNA coding for NaChBac was cloned from a NaChBac expressing *E*. *coli* strain[19] into a pET28a vector (Novagen) between the Nde I and EcoR I restriction sites. As a result, a hexahistidine tag is installed to the N-terminal of NaChBac, linked by a 10 amino acid spacer (Ser-Ser-Gly-Leu-Val-Pro-Arg-Gly-Ser-His). A protease cleavage site (Leu-Val-Pro-Arg-Gly-Ser) is included in the spacer, allowing the hexahistidine (6His-) to be removed by thrombin when required. The cDNA sequence was confirmed by the DNA sequencing service at the University of Alberta Molecular Biology Service Unit.

The expression plasmid is transformed into *E*. *coli* BL21(DE3). A fresh colony was grown in Luria broth (LB) with 30 μg/ml kanamycin for 13–16 hr at 37°C. The cell culture was diluted 100X in Terrific Broth (TB) supplemented with 0.02 M glucose, 15 μg/ml kanamycin and the cells were allowed to grow at 37°C until the optical density at 600 nm (OD600) reached 1.0. The cells were induced by the addition of isopropyl β-D-1-thiogalactopyranoside (IPTG) (final concentration 0.5 mM) and then were allowed to grow for another 4 hr at 37°. The cells were harvested by centrifugation at 9000 ×*g* for 15 min and then the pellets were frozen at -20°C. The pellet was resuspended in 1/40 culture volume of ice-cold lysis buffer (50 mM Tris, 150 mM NaCl, 2.5 mM MgSO_4_, pH 8.0) supplemented with 0.2 mM phenylmethylsulfonyl fluoride (PMSF) and 2 μg/ml DNase I. The cells were lysed using a homogenizer. The sample was then centrifuged at 15,000 ×*g* for 30 min. The supernatant was collected and centrifuged for another 60 min at 140,000 ×*g* to recover the plasma membrane fraction, which was then resuspended to 1/50 of original culture volume in solubilization buffer (50 mM Tris, 150 mM NaCl, 1% n-dodecyl-β-D-maltoside (DDM), pH 8.0) and incubated with gentle shaking at 4°C overnight. The sample was then centrifuged at 140,000 ×*g* for 45 min to remove insoluble impurities. The supernatant, which contains the solubilized NaChBac, was further purified using a HisTrap column (GE Healthcare). The following buffer was used in the affinity purification: wash buffer (50 mM Tris, 150 mM NaCl, 0.1% DDM, 50 mM imidazole, pH 7.4), and elution buffer (50 mM Tris, 150 mM NaCl, 0.1% DDM, 300 mM imidazole, pH7.4). The eluted protein was then concentrated using Amicon Ultra-15 centrifugal filter unit (Millipore, cutoff 10 kDa) to <1/10 of the original volume. The presence and purity of the purified protein was verified by SDS-PAGE (S2 Fig).

### Proteoliposome preparation

Powder form of *E*. *coli* total lipid extract (Avanti lipids) was dissolved in the reconstitution buffer (150 mM NaCl, 500 mM urea, 20 mM Tris, pH 7.4) at a final concentration of 10 mg/ml to form multi-lamella liposome (MLV). 500 mM urea was included in the reconstitution buffer to generate a higher internal osmotic concentration, which would facilitate the fusion of the proteoliposome to the planar bilayer [15]. The lipid solution was then extruded 10 times through a 0.2 μm cellulose acetate membrane syringe filter (Corning) to yield large unilamellar liposomes (LMVs) with diameters around 100 nm. Purified NaChBac was added to the LMV sample in the presence of 1.1% octylglucoside at a varied lipid to protein ratio. The mixture was incubated at room temperature for 1 hr, followed by dilution in the reconstitution buffer to lower the concentration of the detergent to below its critical micelle concentration. The diluted sample was centrifuged at 180, 000 ×*g* and 22°C for 60 min. The liposome sample is recovered by re-suspending the pellet.

### BLM measurement

A BLM workstation equipped with a BC-535 non-inverting amplifier (Warner Instruments, CT, USA), an LPF-8 8-pole Bessel filter (Warner Instruments, CT, USA), and a Digidata 1550 analog-to-digital converter (Molecular Devices, CA, USA) was used for the measurement. Clampex (Molecular Devices, CA, USA) was used for the data acquisition. The planar bilayer was formed by painting a mixture of palmitoyl-oleoyl-phosphatidyl-ethanolamine (POPE) and palmitoyl-oleoyl-phosphatidyl-glycerol (POPG) (mass ratio 3:1, dissolved in decane) across the orifice (diameter 200 μm) of a Delrin sample cup. Protein incorporation was achieved by swelling-driven vesicle fusion. Specifically, the *cis* side of the bilayer was filled with a hypertonic buffer (150 mM NaCl, 20 mM Tris-Cl, pH7.4) compared with the *trans* side (50 mM NaCl, 20 mM Tris-Cl, pH7.4). 0.5 μl of NaChBac proteoliposome was added to the *cis* side and in close proximity to the bilayer. After waiting 5–10 min, a holding potential was applied. Observation of current spikes, or non-zero current, indicated successful fusion of the protein. If the baseline of the current was above 0 pA, it suggested that multiple proteins have been incorporated into the lipid bilayer. If no insertion occured in 10 min, additional proteoliposome sample would be added. After protein insertion, the buffer was exchanged to the desired one using a gravity perfusion setup. A variety of voltage protocols were used and the details are provided in the result of each experiment. All data acquisition was performed at a sampling rate of 12.5 kHz with low-pass filtering at 1 kHz.

### Data analysis

Clampfit (Molecular Devices) and Origin (OriginLab Corporation) were used for data analysis. All raw data were corrected with the electrical interference filter and some were subjected to additional digital filtering using low-pass Bassel filter. The final cut-off frequency for the low-pass filtering is 0.1 to 1 kHz. For macroscopic current recording and the single-channel conductance analysis, the “event detection” function of the Clampfit software was used to detect single opening events and draw event statistics. Generally, 0–100 events could be found, depending on the applied voltage. A current-voltage (I-V) plot was then generated from the event statistics for each condition.

For the blocking effect of QX-314, a set of 25 inactivated current traces from all experimental variables (i.e. no drug, adding a drug, and washout) were averaged and used for baseline subtraction. The average activation trace was obtained after deleting the inactivation traces under each condition and correction for the baseline.

For single channel open probability analysis, the current trace is converted into an all-points histogram, and a Gaussian function is used to fit each peak on the histogram to find the amplitude and the standard deviation for each population. The all-points histogram is then converted into a frequency histogram and normalized to obtain the probability density function. The open and closed probabilities were found by fitting the probability density function with a Gaussian distribution. When N channels are present in the bilayer, the open probability of a single-channel p_o_ can be found by p_a,_ which is the probability that one or more channels open at the same time, by the following equation:

$$p_{o}=1-{\left(1-p_{a}\right)}^{\frac{1}{N}}$$ (1)

For ionic selectivity, the experimental E_rev_ is obtained as the x-intercept of the average current trace. Ionic selectivity, represented by the relative permeability, is then calculated using the following equations:

$$\frac{P_{{Na}^{+}}}{P_{K}^{+}}=\frac{\left[{{Na}^{+}}_{in}\right]}{{{[K}^{+}}_{in}]}\exp\left(-\frac{{FE}_{rev}}{RT}\right)$$ (2)

$$\frac{P_{{Na}^{+}}}{P_{{Ca}^{2+}}}=\frac{4{\left[{Ca}^{2+}\right]}_{out}}{{\left[{Na}^{+}\right]}_{in}\exp\left(\frac{{FE}_{rev}}{RT}\right)(1+\exp\left(\frac{{FE}_{rev}}{RT}\right))}$$ (3)

[A] is the ion concentration expressed in mol/l; F is the Faraday constant with a value of 96,485.3 s·A/mol; E_rev_ is expressed in volts; R is the gas constant 8.314 J/K/mol, and T is the temperature in K.

## Results and discussion

### Reconstitution of NaChBac into planar lipid bilayer

Direct insertion of ion-channels has been challenging, and its incorporation into the planar lipid bilayer is usually achieved by fusion of pre-formed protein vesicles with the planar bilayer [13–15, 20] (S1 Fig). Specifically, unilamellar vesicle was deposited near the bilayer. Swelling of the vesicles that are attached to the planar bilayer, usually induced by the osmotic pressure between the vesicle interior and their bathing solution (*cis* side), and the *trans* side of the membrane, promotes the fusion of the vesicles to the planar bilayer [21]. However, fusion efficiency of vesicles reconstituted with purified ion-selective channels is generally low [21]. The low efficiency is because the swelling was a combined result of water movement from the *trans*-side of the bilayer to the interior of the vesicle and the water/solute efflux from the vesicle to the *cis*-chamber, which requires the channel to be permeable to the solute and open during the fusion process [22]. Swelling-driven fusion was observed to be very low for the NaChBac proteoliposome [15]. After many trials, we have found that an internal buffer with 500 mM urea is sufficient to cause the swelling-driven fusion of NaChBac proteoliposome.

### Macroscopic current recording of full-length NaChBac reconstituted in planar lipid bilayer

#### Unitary channel conductance at varied salt concentration

It was found in an inside-out patch-clamp experiment with NaChBac-overexpressing CHO-K1 cells that the unitary channel conductance of NaChBac is 12±1 pS (8 mM and 130 mM NaCl) [8]. In contrast, BLM characterization of NaChBac in an artificial lipid bilayer formed by POPC/POPE found that NaChBac has a unitary conductance of 120 pS (150 mM NaCl) [15]. In addition, BLM study on the pore-only mutant of the prokaryotic Na_v_ Na_v_SP1 found a conductance value of 40 pS in DPhPC bilayer (200 mM NaCl and 110 mM KCl) [16] or 106 pS in POPC/POPG bilayer (500 mM NaCl) [17]. Multiple sequence alignment of NaChBac and other prokaryotic Na_v_s suggests that they share highly conservative sequence and structure (S1 Fig). The discrepancy among these studies is presumably the result of a different salt condition. To confirm this, we embarked to measure the unitary channel conductance of NaChBac at different salt conditions by BLM and macroscopic current recording.

**Fig 2:**
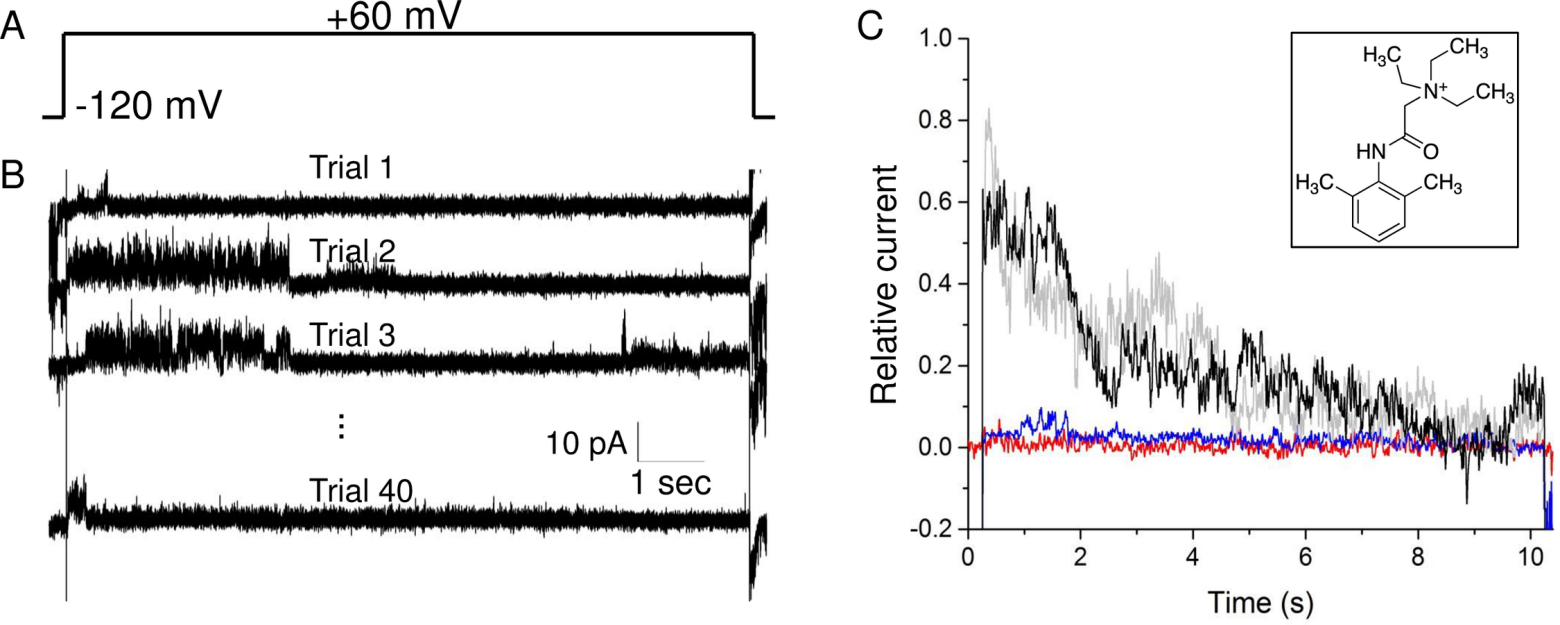
Macroscopic current recording of NaChBac under varied buffer conditions. (A) The voltage step protocol used for the recording. (B) An exemplary current trace when a voltage step of +60 mV was applied under the buffer condition of 150/150 mM NaCl. Note that majority of the channels stay open at -120 mV for the first ~3.4 sec. (C) A magnified view of the dotted blue box in (B) showing opening events of individual channels. (D) The I-V plots of single opening events obtained from macroscopic current recording. (E) Relationship between the unitary conductance and the [NaCl] in the buffer.

Fig 2A shows the voltage protocol used for the recording. Specifically, the membrane bilayer was first held at -120 mV for 5 sec, and then switched to the target voltage step (10 mV intervals from −90 to +60 mV) for 5 sec. Due to the incorporation of multiple proteins and the high activity of the protein, the baseline current is off-zero (Fig 2B). Current from an individual opening channel is determined by analyzing the opening events (n = 30−120) at each voltage step (Fig 2B and 2C). Subsequently, the unitary channel conductance was determined by the zero-voltage slope (Fig 2D).

Using the macroscopic current recording on multiple channels and the analysis of individual opening events, we found that the unitary conductance of NaChBac has a linear relationship with the saline concentration in the range of 30 to 600 mM NaCl (Fig 2E). Attempts to record at a higher [NaCl] failed as the bilayer becomes less stable and it was even more challenging to distinguish individual openings from the noise of the baseline. Thus, the buffer condition may explain the discrepancy observed between the literature values [8, 15], and the literature values and our experimental results in this work.

A linear relationship between conductance and ion-concentration generally suggests a conducting mechanism of independent, constant-field electrodiffusion. However, a weakly coupled multisite/multi-ion mechanism has been suggested for the Na^+^- conducting in Na_v_s based on simulation studies [23–25] and the atomic structure of the permeating pore of prokaryotic Na_v_s [11, 26]. Such a mechanism would demand a complex and saturate conductance-ion-concentration relationship. We have observed a linear relationship between the conductance and the ion concentration in the range of 30–600 mM NaCl, suggesting that NaChBac has a very high saturating concentration that is beyond 600 mM.

In addition, a rectifying effect is clearly observed when a low concentration of NaCl (30 or 150 mM) is present on both sides of the membrane (Fig 2D). That is, the permeation of the Na^+^ is more effective at one voltage polarity compared with the other. The rectifying orientation under these two conditions is opposite to each other, which suggests that the vectorial orientation of the inserted proteins are opposite in these two experiments. The reconstitution of NaChBac into the proteoliposome is a random process hence the overall vectorial orientation of the proteins in the lipid bilayer is also random. It is difficult to judge the presence of rectification at high [Na^+^], since there are not enough single-opening events at either positive holding potentials (e.g. 600 mM NaCl) or both (e.g. 300 mM NaCl).

#### Inhibition of NaChBac by lidocaine N-ethyl bromide

**Fig 3:**
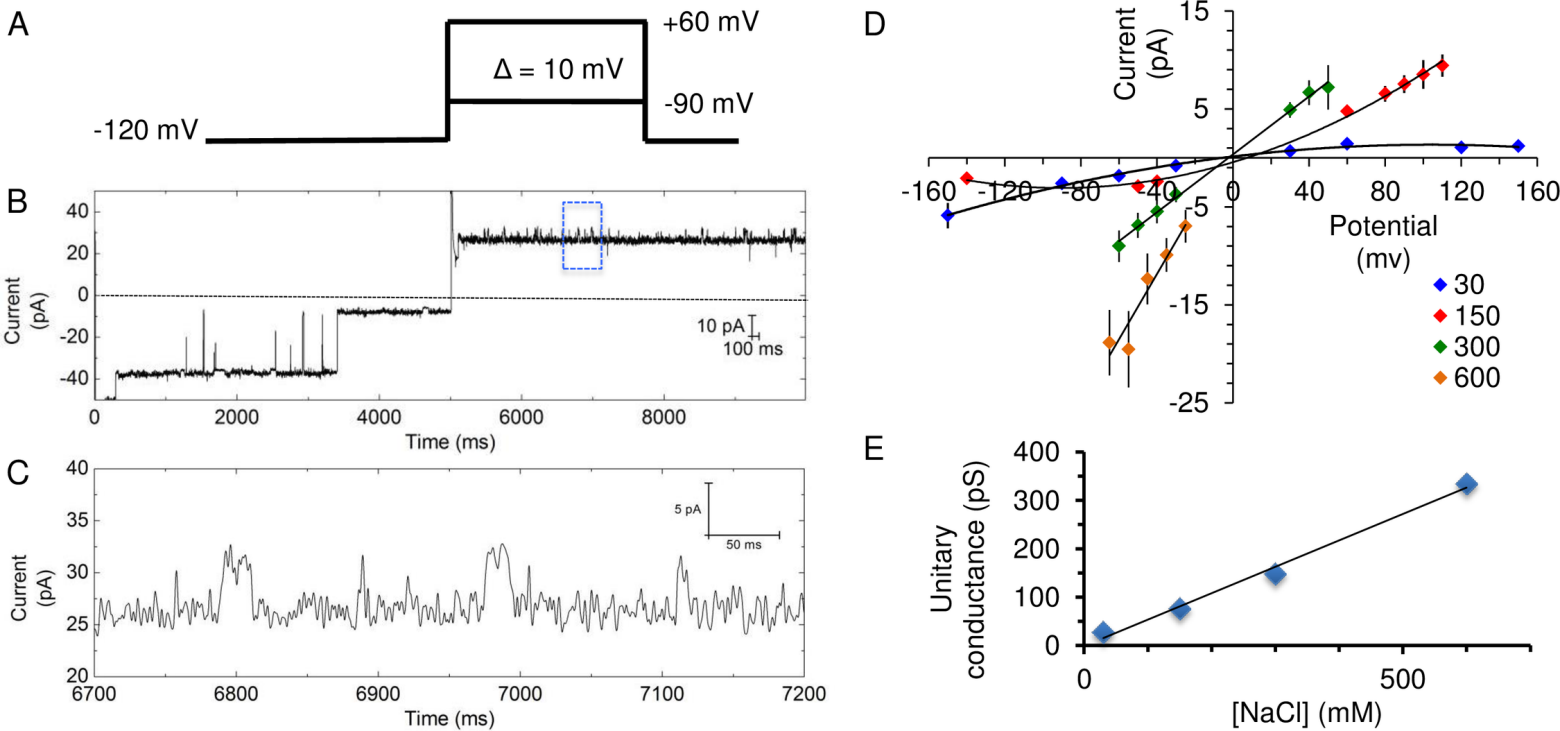
Inhibition of NaChBac by lidocaine N-ethyl bromide. (A) The voltage protocol used in this experiment. (B) 4 out of the 40 current traces from NaChBac channel recording before the drug treatment. (C) Average current trace. Black: before drug application (i.e. control); red: drug applied to the *cis* side of the bilayer; blue: drug applied to both sides; grey: drug removed from the *cis* side. Inset shows the chemical structure of lidocaine N-ethyl bromide.

One disadvantage of BLM is that the membrane proteins may be incorporated into the membrane in either orientation. Some membrane proteins are reported to get inserted into lipid bilayer at a preferred orientation [27], while most proteins are inserted in a random manner. The orientation of the voltage-gated ion channel will prevent proper data interpretation when it displays rectifying effect or when studying the activation at half voltage. To find out the orientation of the inserted proteins, we used lidocaine N-ethyl bromide (QX-314, inset of Fig 3C) [28], which is a membrane impermeable derivative of the archetypal local anesthetic drug lidocaine to selectively block the activity of NaChBac from its intracellular site. It has been reported that QX-314 blocks NaChBac from its intracellular site through a hydrophilic access pathway to an internal binding site [29]. Fig 3 shows the effect of QX-314 on the NaChBac current. In this experiment, NaChBac protein was first incorporated into the lipid bilayer and a voltage protocol, as shown in Fig 3A, was applied. Specifically, the holding potential was first set to -120 mV for 0.5 s before switching to +60 mV for 10 s. The voltage step is repeated 40 times to obtain an average trace (Fig 3B and 3C). The capacitance current was canceled as described in the Methods section. After obtaining successful insertion and initial current recording (black trace of Fig 3C), QX-314 was first added to the *cis* side of the bilayer and the voltage protocol was repeated. It was observed that the current completely dimished (red trace of Fig 3C), suggesting the intracellular side of the inserted NaChBac is located on the *cis* side. Additional drug was then applied to the *trans* side, the current was found to remain at the baseline level (blue trace of Fig 3C). However, after replacing the buffer in the *cis* side with a drug-free solution, the NaChBac activity was recovered to its initial state (gray trace of Fig 3C), further confirming that all the inserted NaChBac protein in this experiment have their intracellular side facing the *cis* side of the bilayer. Hence, NaChBac orientation in the lipid bilayer can be found out by applying the membrane impermeable inhibitor QX-314.

### Single channel behavior of full-length NaChBac

Conventionally, single-channel recording can be achieved by patch-clamping excised membrane from cells heterologously expressing the protein of interest. However, patch-clamp is highly skill-demanding and requires complex instrumental setup [6]. In contrast, single-molecule recording with BLM uses a simpler equipment setup and is less skill-demanding. Recent advances in the knowledge and success on membrane protein purification has overcome the previous hurdle imposed by the difficulties in obtaining purified channel proteins, this greatly facilitates the application of BLM. Here we applied BLM to study the single-channel behavior of full-length NaChBac. As shown in the previous section a single fusion event of NaChBac-containing vesicles to the lipid bilayer introduced multiple copies of NaChBac protein and led to the observation of macroscopic current. In order to obtain single-channel activity, we optimized the lipid-to-protein ratio (LPR) in the proteoliposome. We tested lipid-to-protein ratio (wt/wt) of 1000:1, 2000:1, and 3000:1, and found that using 3000:1 ratio we can relatively readily obtain single-channel insertion.

#### Single-channel conductance and opening probability

**Fig 4:**
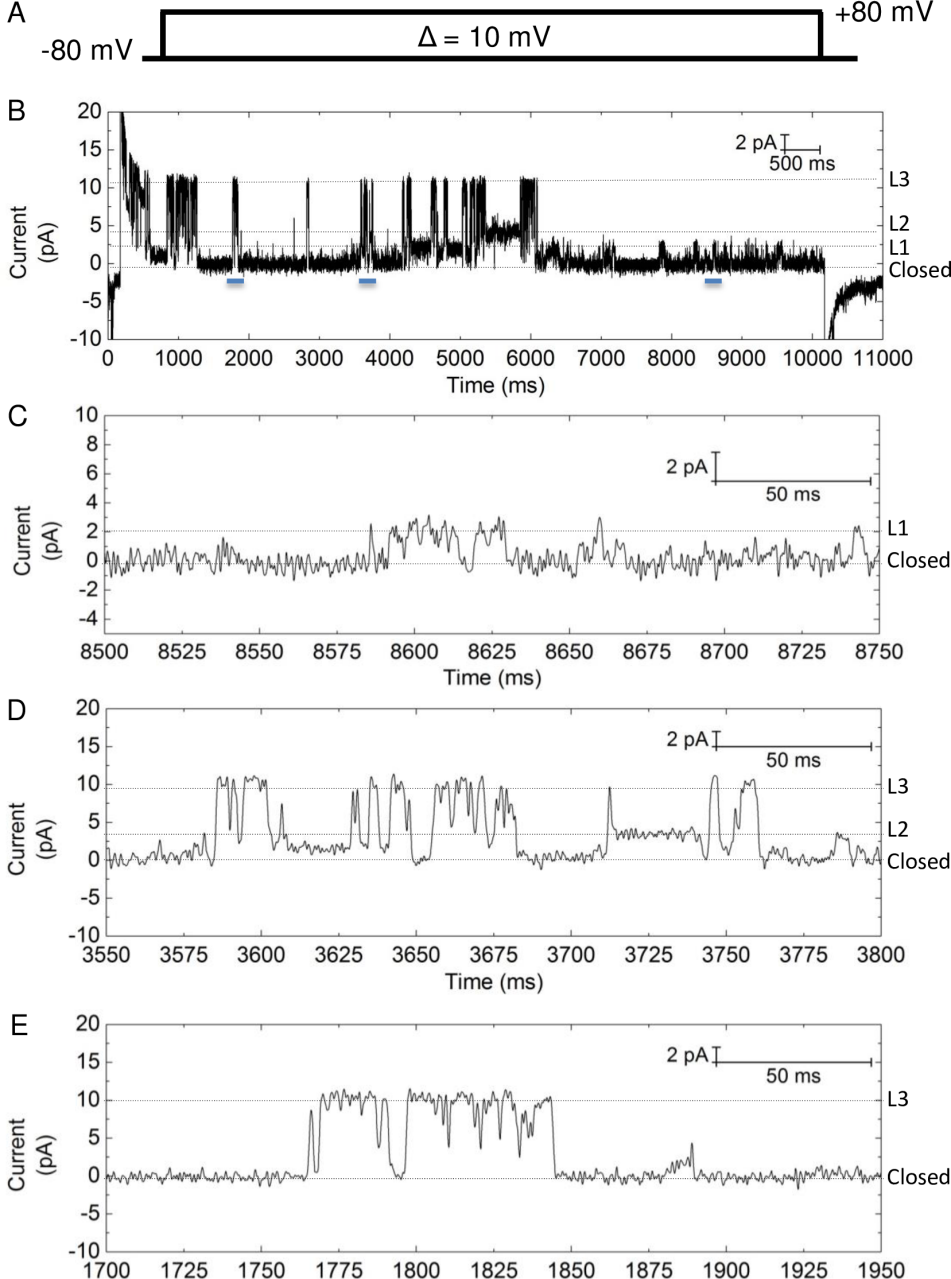
Single-molecule recording of NaChBac for Na^+^ conductance determination. (A) The voltage protocol used for the recording. The channel was first hold at -80 mV for 0.2 sec, followed by a step to an incremented voltage (from -80 mV to 80 mV) for 10 sec and then back to -80 mV. In one experiment the voltage protocol was repeated 5 times and the data pooled for analysis. (B) A representative trace of the recording at +35 mV. Three conducting levels are observed. (C-E). Zoom-in image of (B) as indicated by the blue bars in (B) to show each of the three conducting levels.

**Fig 5:**
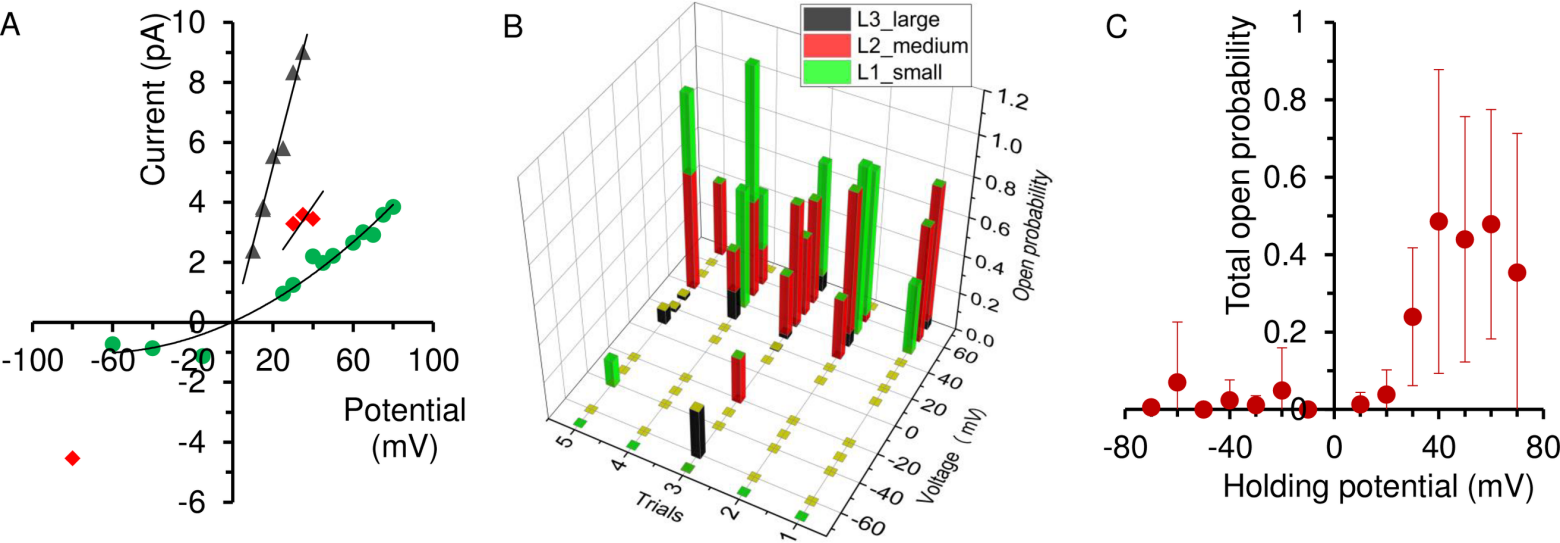
Single-channel conductance and open probability analysis of NaChBac. (A) Representative I-V plot of NaChBac obtained from single-channel recording. Data points are summarized from single-channel event analysis. If no data point displayed, it means that not enough events were found in that particular voltage step. While L1 (red square) opened in both positive and negative step voltage, L3 (green triangle) and L2 (blue diamond) open less frequently at negative step voltage. (B) Distribution of the open probability of the three sub-states at different voltage based on the same experiment of (A). Data were analyzed by all-point current histogram. (C) The total open probability of NaChBac depends on the holding voltage.

After achieving single channel insertion, a voltage step protocol (Fig 4A) was used for current recording under 150/150 mM NaCl condition. Fig 3B shows a representative trace from a recording at which the holding potential was stepped to -35 mV. With single-molecule recording we observed three distinct conducting levels for NaChBac (Fig 4B–4E). By analyzing the individual opening events, the conductance of each of the three levels was calculated as the slope of the I-V plots (Fig 5A). The three sub-conducting levels have the following values: (1) level 1 (L1), 26 ± 6 pS (mean ± SEM, n = 3 independent experiments; same below unless else indicated), (2) level 2 (L2), 93 ± 18 pS, and (3) level 3 (L3), 268 ± 45 pS. L2 is comparable to that found in macroscopic current recording and previous BLM studies (i.e. 120 pS [15]). L1 is comparable to that found in patch-clamp studies of NaChBac (i.e. 12 pS [8]). However, L3, which is the highest conducting sub-state, has not been reported yet. Although transitions among these three levels are observed, for example, the transition from L1 to L3 at time 3630 sec (Fig 4D), the majority of the opening events happen directly from a closed state. These sharp transitions from the closed state to the higher levels of L2 or L3 indicate that they are sub-states of a single protein but not due to the overall recording of multiple proteins. Sub-conducting levels have been found in other ion proteins when studied by BLM. For example, Tom40, a mitochondrial outer membrane protein, was shown to exhibit 4 sub-states with different substrate binding affinities [30]. The voltage-gated K^+^ channel KcsA was also found to exhibit rare and short-lived sub-state when conducting the K^+^ analog Rb^+^ [14]. Our single channel recording thus reveals new channel property of NaChBac that is otherwise difficult to identify in ensemble recording. The three sub-conducting levels observed in this work suggests that the tetrameric NaChBac can exist in different long-lived conducting conformational states.

To find out the orientation of the protein, we added QX-314 to the *trans* side of the bilayer. We were able to observe channel activities upon the treatment of the blocker, as well as the three sub-conducting levels (S2 Fig). This suggests that in this experiment, the intracellular side of NaChBac is located on the *cis* side of the bilayer. However, with the presence of QX-314 in the *trans* side (i.e. extracellular side of the protein), we observed increased channel opening frequency, particularly to L1 and L2 levels, as demonstrated by the availability of more data points in S2 Fig. In contrast, no effect on the ensemble sodium current was found when 200 μM QX-314 was applied to the extracellular site of oocytes overexpressing NaChBac [29].

We then analyzed the opening probability of each of the three sub-conducting levels. Here the opening probability is the accumulated opening duration of a certain sub-conducting level within the tested period. As shown in Fig 5C, opening to L2 and L1 accounts for the majority of the channel opening, and most of the opening events take place at a positive holding potential (i.e. ≥30 mV). The opening probability of L3 is indeed very low. This also explains that the conductance value obtained from the macroscopic recording (76 pS) is similar to the average of L1 and L2 weighted by their probability. There is significant variability in the probability distribution of the three sub-states among trials for the same channel, reflecting the stochastic nature of single-molecule behavior as having been observed in single channel recording of brain sodium channels [10]. From the overall probability plot (Fig 5D), it is clearly shown that the channel opening of the full-length NaChBac is voltage-dependent and strongly favored when the membrane is depolarized. At increased polarization, the protein tends to open at a less conducting sub-state (i.e. L1). Hence, the voltage-dependent activation of the channel is essentially a combined result of the voltage-regulated opening probability, and to a less extent, a voltage-regulated pore conformation.

#### Ionic selectivity of NaChBac reconstituted in planar bilayer

**Fig 6:**
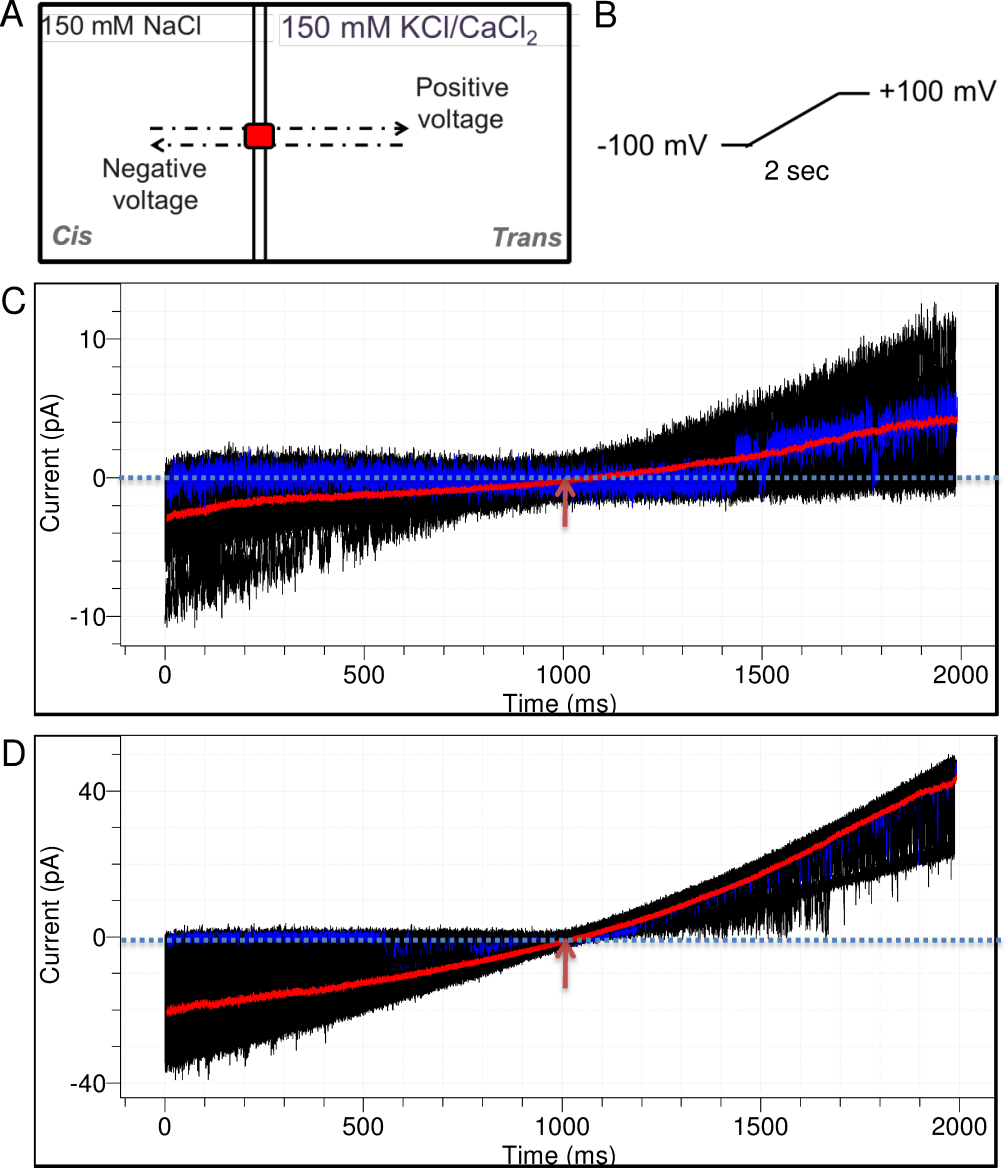
Ionic selectivity of NaChBac at single-molecule BLM recording. (A) Schematic representation of the bi-ionic setup. (B) Schematic representation of the voltage ramp protocol. (C) Current traces under the Na^+^/K^+^ condition. A total of 100 current traces are overlaid and shown in black. The average trace is highlighted in red. Arrowhead indicates the E_rev_. An exemplary trace showing no K^+^ conductance is highlighted in blue. (D) Current traces under the Na^+^/Ca^2+^ condition. Note there are two proteins in this recording. Highlighted traces and arrowhead have the same meaning as (C).

Previous patch-clamp studies suggested that NaChBac is a Na^+^ selective ion channel with a relative permeability of 171 ± 16 for K^+^ and 72 ± 10 for Ca^2+^ [8]. We hereby investigated the ion selectivity behavior of reconstituted NaChBac at the single-molecule level. A bi-ionic set-up as shown in Fig 6A was used. Briefly, the *cis* side of the membrane is filled with a 150 mM NaCl solution while the *trans* side is filled with an MCl solution (M denotes the ion species to be tested) with the same cation concentration. Accordingly, Na^+^ ionic flux dominates the current when a positive voltage is applied, whereas M^+^ flux carries the current when a negative voltage is applied. A voltage ramp protocol, in which the voltage was swept from −100 mV to +100 mV at a speed of 100 mV/s (Fig 6B) was used to find out the reversal potential (E_rev_). Ionic selectivity, as defined by relative permeability, is calculated from the E_rev_ and the salt concentrations using Eqs (2) and (3) in the Method section.

As shown in Fig 6C and 6D, 100 consecutive runs were recorded, and the averaged trace (red traces in Fig 6C and 6D) was used to determine the E_rev_ and thereby the relative permeability. Remarkably, NaChBac shows conductance at negative voltage under both Na^+^/K^+^ and Na^+^/Ca^2+^ bi-ionic conditions. This suggests that, under our testing conditions, NaChBac is permeable to both K^+^ and Ca^2+^. P_Na_/P_K_ was found to be 1.04 ± 0.27 (n = 3) and P_Na_/P_Ca_ 1.58 ± 0.10 (n = 3) (S1 Table). Therefore, NaChBac is permeable to Na^+^ and K^+^ to a similar extent, while slightly more permeable to Na^+^ than Ca^2+^.

However, when examining individual traces, we could find many traces that have no or little conducting at negative voltage whereas channel opening dramatically increases at a positive voltage (for example, blue traces in Fig 6C and 6D). That is, the occurrence of prolonging gating events and the gating frequency are significantly higher at negative voltage than positive holding voltage. Given the ion flow direction at different voltage polarity (Fig 6A), there is Na^+^-mediated current, but less or not K^+^ or Ca^2+^ translocation crossing the channel in these traces.

Therefore, the ionic selectivity of NaChBac is highly stochastic at single-molecular level. The average trace suggests that the channel is permeable to Na^+^, K^+^, and Ca^2+^ to a similar extent in a lipid membrane condition. When translocated, each pair of the three ions generates current flow with similar amplitude (Fig 6C and 6D). However, the high frequency and long duration of gating observed in the K^+^ and Ca^2+^ mediated traces suggested that there is a higher energy barrier and slower translocation for these two ions. Although our selectivity result is different from that obtained in the patch-clamp experiment [8], it is in agreement with the result from single-molecule MD simulation studies [23]. The MD simulation, which is done based on the crystal structure of the Na_v_MS in its activated and open conformation, shows that the pore of the prokaryotic Na_v_ can conduct both Na^+^ and K^+^, but K^+^ appears to be lingering inside the pore much longer and has a lower success rate of translocation [23]. The single-channel recording and analysis here thus provide experimental evidence that protein behavior derived from statistical analysis of a single molecule over time is significantly different from that obtained from ensemble recording of multiple copies of the same molecule.

## Conclusions

Planar lipid bilayer is gaining increasing application for channel protein charaterization [2, 3], and for biosensor development [31]. In this work, purified NaChBac was reconsitituted into a planar lipid bilayer made of POPE and POPG by vesicle fusion. Macroscopic current recording shows that the unitary conductance of the protein has a linear relationship with the buffer’s salt concentration in the range of 30–600 mM. The single-molecule behavior of the full-length protein was examined and found to be highly stochastic. At the single-molelcule level, we were able to observe three sub-states in the protein’s conductance, which indicates the existence of long-lived conducting sub-states. Change in the membrane potential switches on the channel mainly by increasing its opening probability. The reconstituted protein also exhibits selectivity behaviour distinct from ensemble recording, but agrees with single-molecule MD simulation result. At single-molecule level, translocation of any of Na^+^, K^+^, and Ca^2+^ through NaChBac generates similar current flow, while the frequency and probability vary. The results presented here explicitly highlight that single-channel behavior of NaChBac is essentially highly stochastic, which has important implications for the drug design and drug development for Na_v_s.

## Supporting information

S1 Fig Multiple sequence alignment of NaChBac and other prokaryotic Na_v_s. For NaChBac and Na_v_SP1, whose atomic structure is not available, the transmembrane domains were estimated using TMHMM and highlighted in green boxes. For Na_v_Rh, Na_v_Ab and Na_v_MS, the annotation of the transmembrane helices S1 to S6 (highlighted in black boxes) is based on their X-ray crystal structures. The residues corresponding to the selectivity filter are highlighted in red bold text. The four arginine residues that are located in the S4 helix and highly conservative among voltage-gated ion channels are highlighted in grey background.
(TIF)

S2 Fig Schematic representation of osmotic swelling-driven proteoliposome fusion with the planar bilayer. Proteoliposome was added to the proximity of the lipid bilayer (step 1). The vesicle swells due to the osmotic difference between its interior and its surrounding (step 2). Subsequently, the vesicle is fused into the bilayer (step 3), leading to the insertion of the protein (step 4).
(TIF)

S3 Fig Migration pattern of the purified NaChBac on a pseudo-native SDS-PAGE gel. The protein migrates in its monomer form, which has an MW of 33.4 kDa.
(TIF)

S4 Fig Conductance and open probability analysis of the same NaChBac sample in Fig 4 with the presence of 4 mM lidocaine N-ethyl bromide in the *trans* chamber. (TIF)

S1 Table Ionic selectivity of single-molecule NaChBac under a bi-ionic voltage ramp protocol. (DOCX)
