# Supplementary figures and images for: Single-molecule study of full-length NaChBac by planar lipid bilayer recording
Source: PLoS One. 2017 Nov 30;12(11):e0188861. doi: 10.1371/journal.pone.0188861 (PMC5708646; doi:10.1371/journal.pone.0188861)

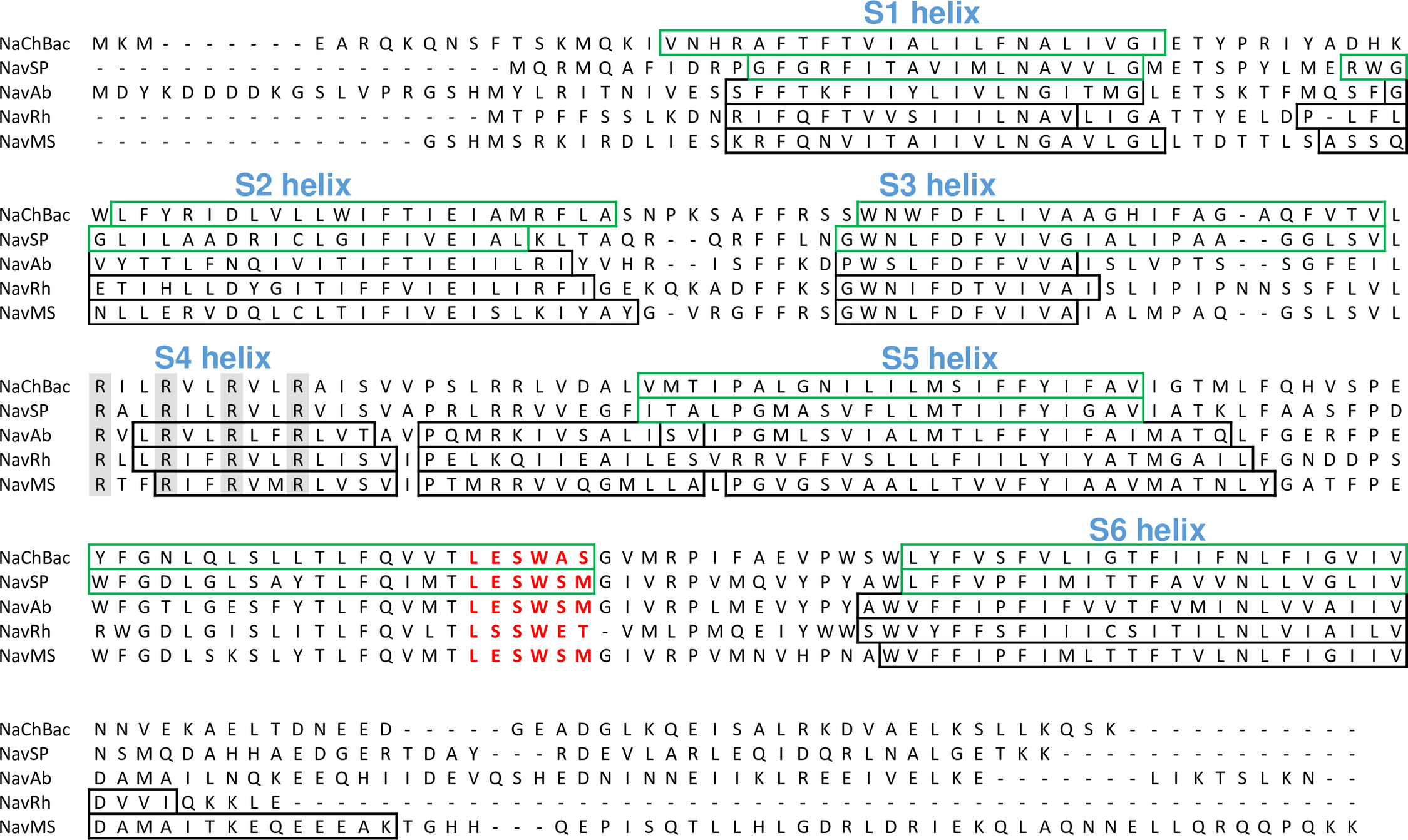

Supplement: S1 Fig — For NaChBac and NavSP1, whose atomic structure is not available, the transmembrane domains were estimated using TMHMM and highlighted in green boxes. For NavRh, NavAb and NavMS, the annotation of the transmembrane helices S1 to S6 (highlighted in black boxes) is based on their X-ray crystal structures. The residues corresponding to the selectivity filter are highlighted in red bold text. The four arginine residues that are located in the S4 helix and highly conservative among voltage-gated ion channels are highlighted in grey background. (TIF) [file pone.0188861.s001.tif]

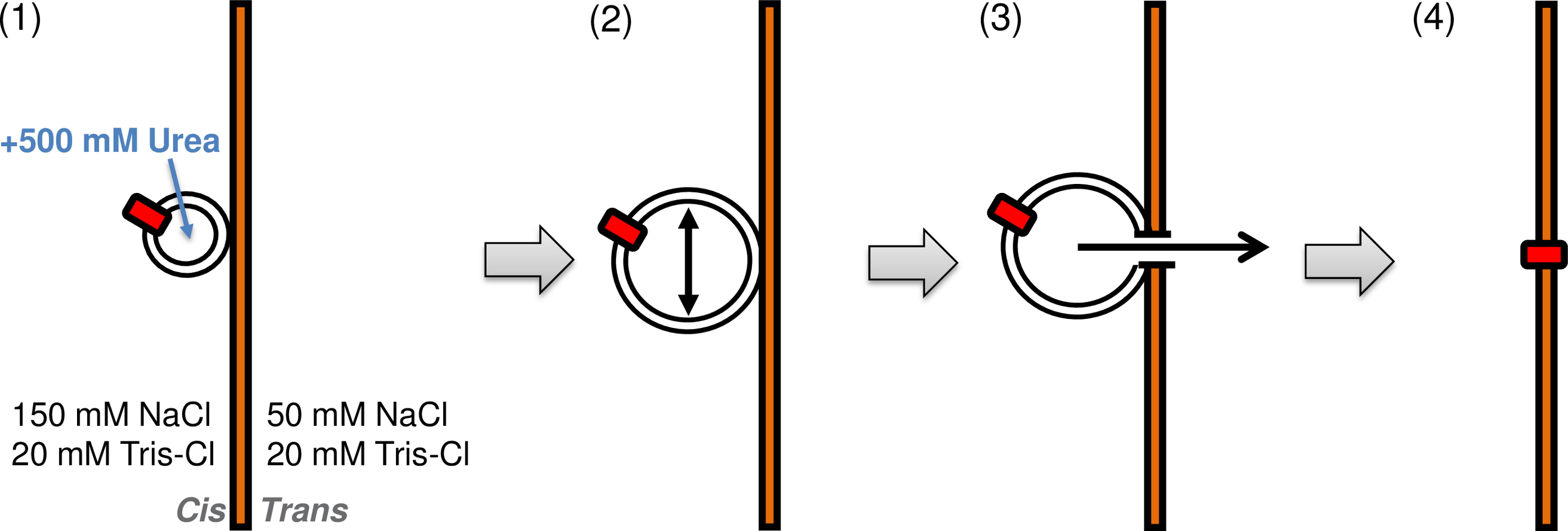

Supplement: S2 Fig — Proteoliposome was added to the proximity of the lipid bilayer (step 1). The vesicle swells due to the osmotic difference between its interior and its surrounding (step 2). Subsequently, the vesicle is fused into the bilayer (step 3), leading to the insertion of the protein (step 4). (TIF) [file pone.0188861.s002.tif]

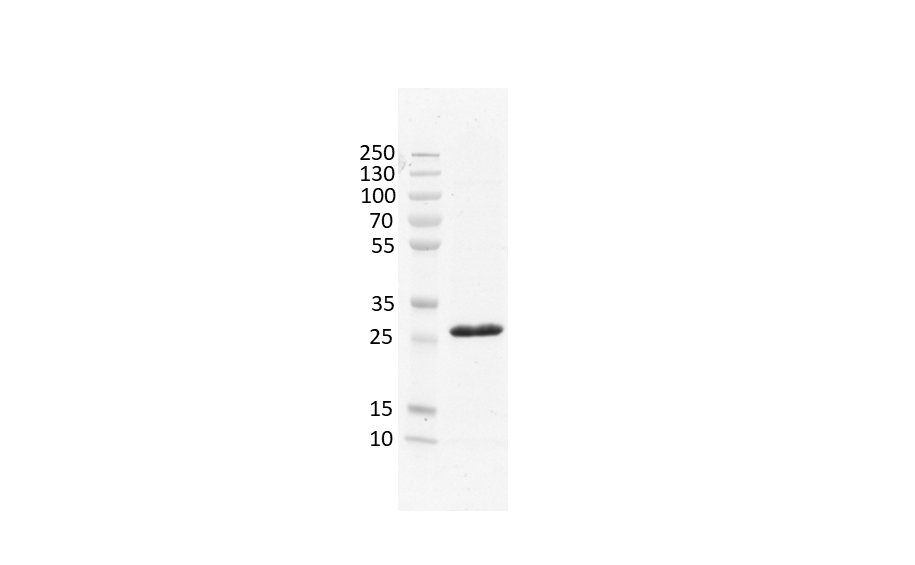

Supplement: S3 Fig — The protein migrates in its monomer form, which has an MW of 33.4 kDa. (TIF) [file pone.0188861.s003.tif]

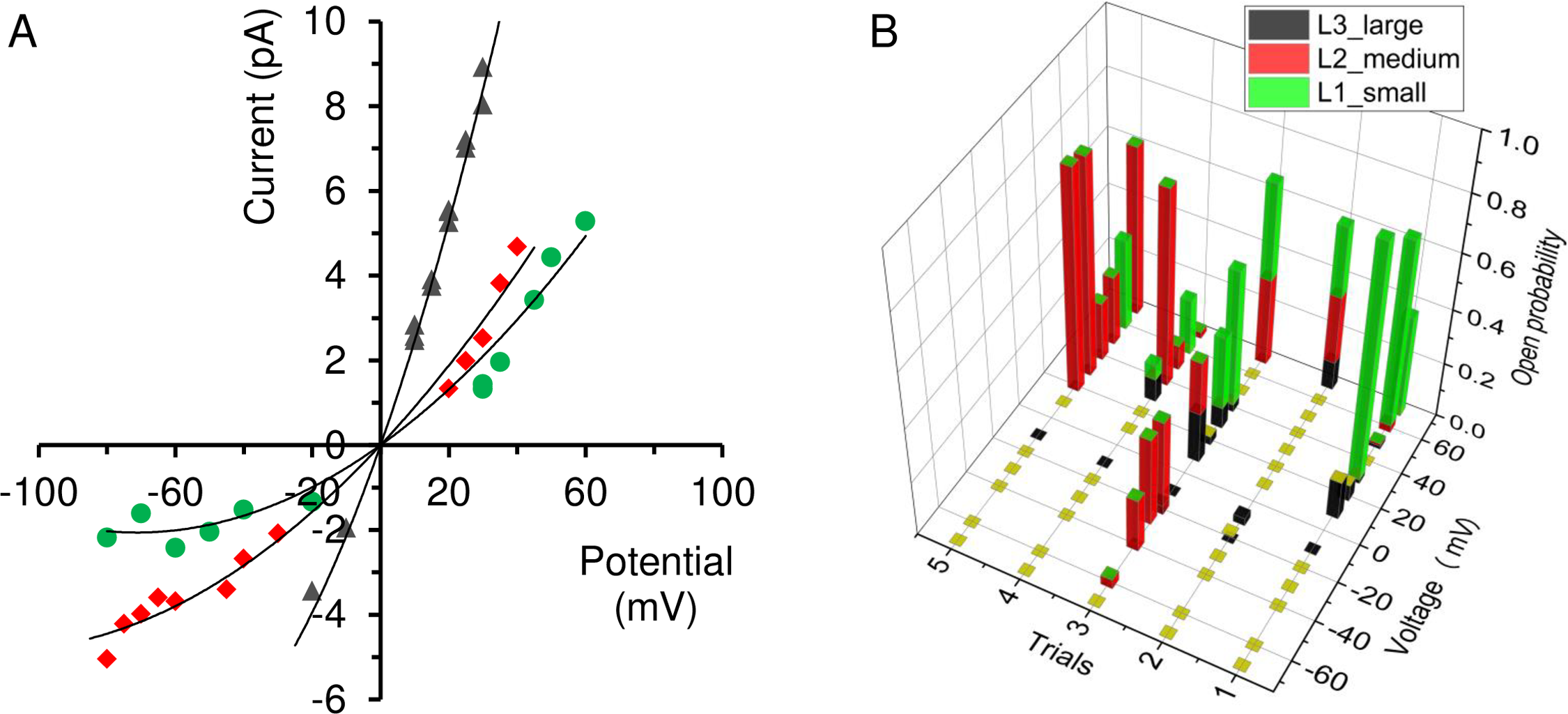

Supplement: S4 Fig — (TIF) [file pone.0188861.s004.tif]
