# Supplementary material for: Single-molecule study of full-length NaChBac by planar lipid bilayer recording
Source: PLoS One. 2017 Nov 30;12(11):e0188861. doi: 10.1371/journal.pone.0188861 (PMC5708646; doi:10.1371/journal.pone.0188861)
Supplement: S1 Table — (DOCX) [file pone.0188861.s005.docx]

**S1 Table.** **Ionic selectivity of single-molecule NaChBac under a bi-ionic voltage ramp protocol.**

| Experiment | Na^+^ v.s. K^+^ | | Na^+^ v.s. Ca^2+^ | |
| --- | --- | --- | --- | --- |
|  | E_v_ (mV) | P_Na+_/P_K+_ | E_v_ (mV) | P_Na+_/P_Ca2+_ |
| 1 | 7.73 | 1.35 | 7.48 | 1.28 |
| 2 | -2.60 | 0.90 | 3.30 | 1.65 |
| 3 | -3.94 | 0.86 | 4.57 | 1.82 |
| Average | 0.40 | 1.04 | 5.11 | 1.58 |
